# The Association of Soluble IGF2R and IGF2R Gene Polymorphism with Type 2 Diabetes

**DOI:** 10.1155/2015/216383

**Published:** 2015-04-02

**Authors:** Suwannee Chanprasertyothin, Wallaya Jongjaroenprasert, Boonsong Ongphiphadhanakul

**Affiliations:** ^1^Research Center, Ramathibodi Hospital, Mahidol University, Rama 6 Road, Bangkok 10400, Thailand; ^2^Department of Medicine, Ramathibodi Hospital, Mahidol University, Rama 6 Road, Bangkok 10400, Thailand

## Abstract

The aim of this study is to investigate the insulin-like growth factor type 2 (IGF2R) gene and circulating soluble IGF2R in relation to type 2 diabetes (T2DM). Six hundred fifty-four subjects without history of diabetes were screened for diabetes by oral glucose tolerance test. In addition, 145 subjects with known diabetes were recruited from a local diabetes clinic. Circulating IGF2R levels were measured by ELISA method; plasma glucose was measured by colorimetric method; insulin levels were determined by chemiluminescent method; IGF2R gene rs416572 was genotyped using real-time PCR. The distributions of IGF2R genotypes were 69.2% CC, 27.8% CT, and 3.0% TT. The C allele was more commonly found in diabetes subjects, with a significant difference (*P* < 0.01). In the presence of the T allele, circulating IGF2R levels were significantly lower (*P* < 0.05). There was no significant difference in other potential confounders including age, sex, and BMI. Only circulating IGF2R, age, and BMI were independently associated with the degree of insulin resistance, as assessed by the HOMA model. It was found that age, sex, and BMI were associated with beta cell function. In conclusion, IGF2R gene polymorphism and circulating IGF2R are associated with T2DM.

## 1. Introduction

The insulin-like growth factor (IGF) system and its binding proteins play important roles in a number of biological processes, including growth and development as well as energy homeostasis. There is increasing evidence that the IGF system is a contributing factor in the pathogenesis of diabetes [1]. With multiple ligands, binding proteins, and receptors, the IGF system is complex; the biological effects are the results of the interactions among the components in the system. It is of note that rather than mediating intracellular signaling transduction elicited by IGF2, the IGF type 2 receptor (IGF2R) acts as a IGF2 scavenger. IGF2R is implicated in diverse biological processes including carcinogenesis [2], glucose homeostasis [3], and HIV replication [4]. With regard to glucose homeostasis, genetic variants in the IGF2R gene have been shown to be associated with both type 1 [5] and type 2 diabetes (T2DM) [6]. Furthermore, a soluble form of IGF2R exists in the circulation and has been found to be related to body size in term infants [7]. Taken together, this suggests the importance of IGF2R in the pathogenesis of disorders related to glucose and energy metabolism. However, whether circulating IGF2R is determined by IGF2R gene variants and how soluble IGR2R is related to diabetes in adults are currently unknown. It is therefore the purpose of the present study to investigate the IGF2R gene and circulating soluble IGF2R in relation to T2DM in adults.

## 2. Materials and Methods

### 2.1. Subjects

Subjects consisted of 654 women and men without known history of diabetes who were invited for a screening of diabetes. A 75 gm oral glucose tolerance test (OGTT) was performed in the morning after an overnight fast. According to American Diabetes Association criteria [8], subjects were divided into three groups: subjects with normal glucose tolerance, IFG/IGT (impaired fasting glucose/impaired glucose tolerance), and subjects with diabetes (297, 260, and 97, resp.). In addition, 145 subjects with known diabetes were recruited from a local diabetes clinic. The study was approved by the ethical review board of Ramathibodi Hospital, and volunteers signed informed consent prior to the study.

### 2.2. DNA Pooling Preparation

DNA was extracted from peripheral blood samples by conventional phenol-chloroform method. DNA samples were diluted in TE buffer (0.1 mM EDTA, 10 mM Tris HCl, and pH 8.0) and quantified using fluorometry (Hoefer DyNA Quant 200; Hoechst dye) to a target concentration of 100 ng/*μ*L and then to 50 ng/*μ*L. DNA pools were constructed separately for subjects with and without diabetes by combining the 50 ng/*μ*L DNA in equal volume. Each pool was purified and again quantified exactly to 50 ng/*μ*L.

### 2.3. Allele Frequency Estimation from DNA Pools Using SNP Genotyping Microarray

Subjects were divided into four groups: (1) diabetes subjects with body mass index (BMI) of less than 25 kg/m^2^; (2) diabetes subjects with BMI of more than or equal to 25 kg/m^2^; (3) normal subjects with BMI of less than 25 kg/m^2^; (4) normal subjects with BMI of more than or equal to 25 kg/m^2^. Each group consisted of 100 subjects for performing pooled DNA samples. Four pooled DNA samples were then genotyped in triplicate using GeneChip Mapping 500 K arrays (Affymetrix, Santa Clara, CA, USA) following the manufacturer's protocol. Briefly, 250 ng of DNA sample from each pool was restricted by Nsp I or Sty I restriction endonuclease before ligation. The diluted ligated DNA was amplified by PCR. Purified PCR products were fragmented, labeled, and then hybridized. Estimated allele frequencies and odds ratios for each SNP were calculated by averaging hybridization intensity signals from the arrays. SNP rs416572 possessed the highest estimated odds ratio and was selected for individual genotyping.

### 2.4. Individual SNP Genotyping

Data from pooled DNA SNP screening revealed that allele frequency of an intronic C/T SNP rs416572 in the IGF2R gene is different between subjects with and without diabetes. Individual genotyping of all subjects was performed using real-time PCR (TaqMan MGB probes); 20 ng of DNA was added into the PCR reaction, consisting of TaqMan Universal Master Mix (1x) and TaqMan MGB probes for intronic C/T SNP rs416572 (1x) in a total volume of 20 *μ*L. The real-time PCR reaction protocol was 10 min at 95°C, 40 cycles of 15 sec at 95°C, and 1 min at 60°C using Applied Biosystems 7500 Real-Time PCR System (Life Technologies, Carlsbad, CA, USA).

### 2.5. Biochemical Measurement

Circulating IGF2R levels were measured by ELISA method (CUSABIO, Wuhan, China). Plasma glucose was measured by colorimetric method. Insulin levels were determined by chemiluminescent method.

### 2.6. Statistical Analysis

Data were expressed as mean ± SD. The differences of variables were determined by ANOVA and Student's *t*-test. The relationship between variables was determined by multiple logistic regression. Statistical significance was assigned at *P* < 0.05.

## 3. Results

Table 1 demonstrates the clinical characteristics of the study population. Screening of 654 subjects with no previous history of diabetes revealed diabetes and IFG/IGT in 97 and 260, respectively. Across the glucose tolerance status in subjects without previous diabetes, there were differences in weight, BMI, fasting plasma glucose, and 2 h plasma glucose, as shown in Table 1.

Screening for SNP in IGF2R with pooled DNA samples revealed that rs416572 is likely to have different allele frequency between diabetic and nondiabetic subjects. Individual genotyping was then performed and the genotype distribution was 69.2% CC, 27.8% CT, and 3.0% TT. The genotype distribution conformed to the Hardy-Weinberg equilibrium. When comparing the genotype distributions according to the presence or absence of diabetes, it was found that the distributions were significantly different with the C allele more commonly found in subjects with diabetes (*P* < 0.01), as shown in Table 2.

Logistic regression was then performed to control for other confounding factors for diabetes including age, gender, and BMI. As shown in Table 3, each C allele of the IGF2R polymorphism was associated with increasing risk of T2DM independently of age, gender, and BMI. To explore potential mechanisms underlying the association between IGF2R genotype and diabetes, analyses of circulating IGF2R levels were performed. In order to exclude the potential confounding effect of longstanding diabetes and diabetes treatments on circulating IGF2R, only subjects with newly diagnosed diabetes (*n* = 97) were studied together with subjects having normal glucose tolerance and IFG/IGT. As shown in Table 4, in the presence of the T allele (genotypes CT and TT), circulating IGF2R levels were significantly lower (*P* < 0.05). There was no significant difference in other potential confounders including age, sex, and BMI. In a multiple regression model, the effect of BMI did not reach statistical significance (*P* = 0.98) while that of the IGF2R allele almost reached statistical significance (*P* = 0.09).

We further explored the influence of both IGF2R gene polymorphism and IGF2R levels on insulin resistance as assessed by the homeostatic model assessment (HOMA) model. As shown in a linear regression model with age, sex, BMI, IGF2R gene polymorphism, and circulating IGF2R as independent variables, only circulating IGF2R, age, and BMI were independently associated with the degree of insulin resistance. In a similar model for HOMA-B it was found that age, sex, and BMI were associated with beta cell function, while the IGF2R gene and circulating IGF2R had no effect (Table 5).

## 4. Discussion

T2DM is largely genetically determined. A number of genome-wide association studies have implicated variants of a number of genes in the pathogenesis of T2DM [9, 10]. Most of these genes encode proteins involved in the proliferation and function of pancreatic beta cells. With regard to the IGF system, only insulin-like growth factor-binding protein 2 (IGFBP2) [11–13] has been implicated in genome-wide association studies in various populations so far, although not without discrepant results [14]. The genetic variant in IGF2BP2 affects first-phase insulin secretion [15] and may be the underlying basis for the influence of IGF2BP2 polymorphism on the susceptibility to T2DM. In the present study, we have identified a genetic variant in IGF2R, another protein in the IGF system, as being associated with T2DM in Thais. A previous study of IGF2R as a susceptibility gene for T2DM has similarly implicated an insertion/deletion variant in the 3′UTR region of IGF2R [6]. The insertion/deletion polymorphism is likely to result in the change in IGF2R expression [16]. Although the previously reported insertion/deletion polymorphism was not genotyped, the SNP investigated in the present study is located in more than 50,000 nucleotides from the 3′UTR, so the possibility of its being in linkage disequilibrium with the 3′UTR insertion/deletion polymorphism is small.

Not unlike IGF1, IGF2R is expressed ubiquitously. Rather than mediating the effect of IGF2, IGF2R modulates the abundance of IGF1 by leading IGF1 through lysosomal degradation after its binding to IGF2R [4]. Although IGF2R is mainly a membrane receptor, its soluble form exists in the circulation. However, the physiologic role of soluble IGF2R is not entirely clear. In a recent study, it was suggested that soluble IGF2R may play a role in angiogenesis by blocking plasminogen activation [17]. In the present study we demonstrated that soluble IGF2R levels were associated with IGF2R genotype, and it is likely that IGF2R gene polymorphism may influence diabetes-related phenotypes partly through its association with the availability of soluble IGF2R. Indeed, the association between IGF2R genotype and diabetes-related phenotypes including age, gender, and BMI became less apparent or disappeared after controlling for circulatory IGF2R. Previous studies investigating soluble IGF2R are scarce. Our finding, however, is in keeping with a recent study showing similar relationships between soluble IGF2R and glucose homeostasis and serum lipids [18].

How soluble IGF2R is related to diabetes is unclear. Soluble IGF2R could directly affect susceptibility to type 2 diabetes, or indirectly through growth hormone (GH) and IGF1. The GH/IGF1 system is very complex, and the influence of each single component in the system on biological processes of interest cannot be easily teased out. IGF2R is mainly a membrane receptor without intracellular signaling function. The major effect of IGF2R is to act as a scavenger receptor for IGF2 and IGF1 by the internalization of IGFs to lysosomes [19]. It is unknown at present how soluble IGF2R is related to membrane-bound IGF2R and what the biological function of soluble IGF2R is. IGF1, however, is believed to be the predominant effector of the GH/IGF1 system, although a number of IGF-binding proteins have recently been established to have direct biological functions of their own. Again, there has been no study on the relationship between soluble IGF2R and these well-established players in the IGF system. However, a more recent meta-analysis of GWAS for type 2 diabetes studies in African Americans has found a novel type 2 DM locus near the insulin and IGF2 genes [20]. Moreover, IGF2 overexpression in transgenic mice leads to islet hyperplasia [21] and IGF2 deficiency in the Goto-Kakizaki rat leads to beta cell mass anomaly [22]. Taken together with the association of IGF2R gene polymorphism and soluble IGF2R to diabetes in the present study, further investigation to elucidate how soluble IGF2R fits in the complex system of IGF and how IGF2 together with IGF2R influence the susceptibility to type 2 DM is warranted.

## Figures and Tables

**Table 1:** Clinical characteristics of the study population.

	Normal (*n* = 297)	IFG/IGT (*n* = 260)	Unknown DM (*n* = 97)	*P* value^*^	Known DM (*n* = 145)	*P* value^**^
Age (year)	61.7 ± 8.1	63.0 ± 7.8	63.0 ± 6.7	NS	60.3 ± 11.0	NS
% female	82.9	83.9	87.8	NS	65.6	<0.01
Weight (kg)	57.9 ± 10.1	61.3 ± 9.8	65.1 ± 10.1	<0.01	64.0 ± 13.1	<0.01
Height (cm)	154.5 ± 7.2	154.1 ± 6.8	153.9 ± 6.0	NS	157.1 ± 9.6	<0.01
BMI (kg/m^2^)	24.2 ± 3.6	25.8 ± 3.6	27.6 ± 4.4	<0.01	26.1 ± 6.5	<0.01
Fasting plasma glucose (mg/dL)	90.2 ± 6.0	100.5 ± 8.7	129.5 ± 36.9	<0.01	148.4 ± 47.3	<0.01
2 hr glucose (mg/dL)	110.6 ± 16.4	146.5 ± 26.5	263.1 ± 55.1	<0.01	—	—
HOMA-IR	1.5 ± 0.8	2.3 ± 1.4	4.6 ± 3.3	<0.01	5.8 ± 6.3	<0.01
HOMA-B	95.2 ± 57.5	90.4 ± 44.4	95.8 ± 119.8	NS	76.5 ± 5.0	<0.01

^∗^Comparison among subjects with no previous history of diabetes.

^**^Comparison between normal and known DM subjects.

DM = diabetes mellitus; HOMA = homeostatic model assessment; NS = not significant.

**Table 2:** Genotype distribution of rs416572 in relation to the presence of diabetes.

IGF2R genotype	Non-DM (*n* = 557)	DM (*n* = 242)
CC	366 (65.7%)	177 (73.1%)
CT	169 (30.3%)	63 (26.0%)
TT	22 (4.0%)	2 (0.8%)

**Table 3:** Factors associated with diabetes from logistic regression analysis.

	Odds ratio	95% CI	*P* value
IGF2R genotype (per each C allele)	1.45	1.06–1.96	<0.05
Age (year)	0.99	0.98–1.01	NS
Female gender	0.54	0.37–0.78	<0.01
BMI (kg/m^2^)	1.10	1.06–1.14	<0.01

**Table 4:** The differences of variables between groups with and without the T allele.

	IGR2R-CT/TT	IGF2R-CC	*P* value
IGF2R levels (pg/mL)	10,964.9 ± 413.2	12,029.0 ± 327.0	<0.05
Age (years)	63.0 ± 0.5	62.1 ± 0.4	NS
% female	83.1	84.3	NS
BMI (kg/m^2^)	25.0 ± 0.3	25.2 ± 0.2	NS

**Table 5:** The association between the HOMA models, circulating IGF2R, and the IGF2R gene.

	HOMA-IR		HOMA-B	
	Beta	*P* value	Beta	*P* value
IGF2R levels (pg/mL)	−0.07	<0.05	0.03	NS
IGF2R gene (per each C allele)	0.02	NS	−0.00	NS
Age (year)	−0.08	<0.05	0.10	<0.05
Female gender	−0.06	NS	0.11	<0.05
BMI (kg/m^2^)	−0.53	<0.05	0.30	<0.05

## References

[1] Holt R. I. G., Simpson H. L., Sönksen P. H. (2003). The role of the growth hormone-insulin-like growth factor axis in glucose homeostasis. *Diabetic Medicine*.

[2] Martin-Kleiner I., Gall Troselj K. (2010). Mannose-6-phosphate/insulin-like growth factor 2 receptor (M6P/IGF2R) in carcinogenesis. *Cancer Letters*.

[3] Ong K. K. L., Dunger D. B. (2001). Developmental aspects in the pathogenesis of type 2 diabetes. *Molecular and Cellular Endocrinology*.

[4] Suh H.-S., Cosenza-Nashat M., Choi N. (2010). Insulin-like growth factor 2 receptor is an IFN*γ*-inducible microglial protein that facilitates intracellular HIV replication: implications for HIV-induced neurocognitive disorders. *The American Journal of Pathology*.

[5] McCann J. A., Xu Y. Q., Frechette R., Guazzarotti L., Polychronakos C. (2004). The insulin-like growth factor-II receptor gene is associated with type 1 diabetes: evidence of a maternal effect. *The Journal of Clinical Endocrinology & Metabolism*.

[6] Villuendas G., Botella-Carretero J. I., López-Bermejo A. (2006). The ACAA-insertion/deletion polymorphism at the 3′ UTR of the IGF-II receptor gene is associated with type 2 diabetes and surrogate markers of insulin resistance. *European Journal of Endocrinology*.

[7] Ong K., Kratzsch J., Kiess W., Costello M., Scott C., Dunger D. (2000). Size at birth and cord blood levels of insulin, insulin-like growth factor I (IGF-I), IGF-II, IGF-binding protein-1 (IGFBP-1), IGFBP-3, and the soluble IGF-II/mannose-6-phosphate receptor in term human infants. The ALSPAC Study Team. Avon Longitudinal Study of Pregnancy and Childhood. *Journal of Clinical Endocrinology and Metabolism*.

[8] American Diabetes Association (2013). Standards of medical cares in diabetes—2013. *Diabetes Care*.

[9] Frayling T. M. (2007). Genome-wide association studies provide new insights into type 2 diabetes aetiology. *Nature Reviews Genetics*.

[10] McCarthy M. I. (2010). Genomics, type 2 diabetes, and obesity. *The New England Journal of Medicine*.

[11] Grarup N., Rose C. S., Andersson E. A. (2007). Studies of association of variants near the HHEX, CDKN2A/B, and IGF2BP2 genes with type 2 diabetes and impaired insulin release in 10,705 Danish subjects: validation and extension of genome-wide association studies. *Diabetes*.

[12] Wu Y., Li H., Loos R. J. F. (2008). Common variants in *CDKAL1*, *CDKN2A*/B, *IGF2BP2*, *SLC30A8*, and *HHEX/IDE* genes are associated with type 2 diabetes and impaired fasting glucose in a Chinese Han population. *Diabetes*.

[13] Chauhan G., Spurgeon C. J., Tabassum R. (2010). Impact of common variants of *PPARG, KCNJ11, TCF7L2, SLC30A8, HHEX, CDKN2A, IGF2BP2*, and *CDKAL1* on the risk of type 2 diabetes in 5,164 Indians. *Diabetes*.

[14] Lee Y.-H., Kang E. S., Kim S. H. (2008). Association between polymorphisms in *SLC30A8, HHEX, CDKN2A/B, IGF2BP2, FTO, WFS1, CDKAL1, KCNQ1* and type 2 diabetes in the Korean population. *Journal of Human Genetics*.

[15] Groenewoud M. J., Dekker J. M., Fritsche A. (2008). Variants of *CDKAL1* and *IGF2BP2* affect first-phase insulin secretion during hyperglycaemic clamps. *Diabetologia*.

[16] Lv K., Guo Y., Zhang Y., Wang K., Jia Y., Sun S. (2008). Allele-specific targeting of hsa-miR-657 to human IGF2R creates a potential mechanism underlying the association of ACAA-insertion/deletion polymorphism with type 2 diabetes. *Biochemical and Biophysical Research Communications*.

[17] Leksa V., Loewe R., Binder B. (2011). Soluble M6P/IGF2R released by TACE controls angiogenesis via blocking plasminogen activation. *Circulation Research*.

[18] Jeyaratnaganthan N., Højlund K., Kroustrup J. P. (2010). Circulating levels of insulin-like growth factor-II/mannose-6-phosphate receptor in obesity and type 2 diabetes. *Growth Hormone & IGF Research*.

[19] Liu Q., Yan H., Dawes N. J., Mottino G. A., Frank J. S., Zhu H. (1996). Insulin-like growth factor II induces DNA synthesis in fetal ventricular myocytes in vitro. *Circulation Research*.

[20] Ng M. C., Shriner D., Chen B. H. (2014). Meta-analysis of genome-wide association studies in African Americans provides insights into the genetic architecture of type 2 diabetes. *Plos Genetics*.

[21] Petrik J., Pell J. M., Arany E. (1999). Overexpression of insulin-like growth factor-II in transgenic mice is associated with pancreatic islet cell hyperplasia. *Endocrinology*.

[22] Calderari S., Gangnerau M.-N., Thibault M. (2007). Defective IGF2 and IGF1R protein production in embryonic pancreas precedes beta cell mass anomaly in the Goto-Kakizaki rat model of type 2 diabetes. *Diabetologia*.

